# Biochemical, Antioxidant Properties and Antimicrobial Activity of Steno-Endemic *Origanum onites*

**DOI:** 10.3390/microorganisms11081987

**Published:** 2023-08-02

**Authors:** Kerem Canli, Mustafa Eray Bozyel, Dilay Turu, Atakan Benek, Ozcan Simsek, Ergin Murat Altuner

**Affiliations:** 1 Department of Biology, Faculty of Science, Dokuz Eylül University, Izmir 35390, Türkiye; 2 Fauna and Flora Research and Application Center, Dokuz Eylül University, Izmir 35390, Türkiye; 3 Department of Biology, Graduate School of Natural and Applied Science, Dokuz Eylül University, Izmir 35390, Türkiye; 4 Department of Biology, Graduate School of Natural and Applied Sciences, Kastamonu University, Kastamonu 37150, Türkiye; 5 Department of Forestry, Yenice Vocational School, Çanakkale Onsekiz Mart University, Çanakkale 17950, Türkiye; 6 Department of Biology, Faculty of Science, Kastamonu University, Kastamonu 37150, Türkiye

**Keywords:** *Origanum onites*, antimicrobial activities, antioxidant activity

## Abstract

*Origanum onites* (Lamiaceae) is an Eastern Mediterranean plant that is widely used in Turkish traditional medicine. This study aimed to investigate the biochemical composition, antimicrobial activity, and antioxidant potential of *O. onites*. In this study, the biochemical composition of the *O. onites* ethanol extract (OOEt) was analyzed using GC-MS. The antimicrobial activity was investigated using a disk diffusion test and determining minimum inhibitory concentrations (MIC) against 30 microorganism strains, including 28 bacteria (some multidrug-resistant) and 2 fungi. Additionally, the antioxidant activity was evaluated using the DPPH method. The main component identified was carvacrol. OOEt demonstrated antimicrobial activity against a wide range of tested microorganism strains. OOEt displayed the highest activity against *E. faecium* (a Gram-positive bacterium) at 100 µL with a 52 mm inhibition zone. Additionally, *P. aeruginosa* DSMZ 50071 and *P. fluorescens* P1, which are Gram-negative bacteria, were the most sensitive strains with a 24 mm inhibition zone in 100 µL of OOEt. The data obtained from *A. baumannii* (a multidrug-resistant strain) is particularly striking, as higher activity was observed compared to all positive controls. All tested fungal strains showed more effective results than positive controls. The antioxidant activity of OOEt was found to be stronger than that of the positive control, ascorbic acid. This study determined that *O. onites* has significant antimicrobial and antioxidant potential.

## 1. Introduction

Throughout human history, medicinal plants have played a crucial role in the prevention and treatment of various diseases [1]. The increasing prevalence of diseases in modern times has led to a renewed interest in the medicinal properties of these plants for therapeutic purposes [2]. One such plant with notable antimicrobial and antioxidant properties is *Origanum onites*, commonly known as Turkish oregano.

The use of medicinal plants dates back to ancient civilizations, where they were employed to treat a wide range of ailments [3]. Traditional systems of medicine, such as Ayurveda, Traditional Chinese Medicine, and Unani, have relied heavily on the therapeutic properties of plants [4]. Today, many modern pharmaceuticals are derived from plant sources, highlighting the continued importance of medicinal plants in healthcare [1].

The rise in antibiotic-resistant pathogens has become a significant global health concern [5]. As a result, researchers are increasingly exploring the antimicrobial properties of medicinal plants as potential alternatives to conventional antibiotics [6]. Numerous studies have demonstrated the effectiveness of plant extracts and essential oils against a variety of pathogenic microorganisms, including bacteria, fungi, and viruses [7].

The antioxidant properties of medicinal plants are also of great interest due to their potential role in preventing chronic diseases such as cancer, cardiovascular disease, and neurodegenerative disorders [8]. Oxidative stress, caused by an imbalance between the production of reactive oxygen species (ROS) and the body’s antioxidant defenses, has been implicated in the pathogenesis of these diseases [9]. Plant-derived antioxidants, such as flavonoids, phenolic acids, and terpenoids, can help neutralize ROS and protect cells from oxidative damage [8].

There are about 23 species and 32 taxa related to the genus *Origanum* (Lamiaceae) in Turkey [10,11]. In Anatolia, members of the genus *Origanum* are often used as culinary herbs, spices, and herbal tea, and are called kekik [10,12,13,14]. *O. onites* is called by names such as ari kekik, bilya kekik, bilyali kekik, incir kekigi, izmir kekigi, kirkbas kekik, tokali kekik, and yemis kekigi in Turkish [15,16,17]. *O. onites* is a steno-endemic taxon with a narrow distribution area covering only the Eastern Mediterranean region [18]. The leaves of this plant are widely used in traditional medicine due to their antimicrobial and antioxidant properties [19].

The antimicrobial properties of *O. onites* are attributed to its bioactive compounds, such as phenolic acids and terpenoids, which demonstrate effectiveness against various pathogenic microorganisms [20]. Numerous studies have shown that extracts of the plant inhibit the growth of bacteria and fungi and, in some cases, even kill them [21]. In particular, the antimicrobial properties of *O. onites* have enabled its use as a natural preservative in the food industry [22].

The antioxidant properties of *O. onites* are significant due to their ability to neutralize free radicals [19]. Free radicals are molecules that can cause cellular damage and aging. The antioxidant properties of *O. onites*, attributed to its high content of flavonoids, phenolic acids, and terpenoids, may play a potential role in preventing cancer, heart disease, and other chronic diseases [23].

Among the medicinal uses of *O. onites* is the treatment of various ailments, such as respiratory infections, digestive system issues, pain, and inflammation [20]. Additionally, due to its antioxidant properties, the plant can also be used for skin health [23].

According to previous research, *O. onites* has been found to contain a variety of significant essential oils, including carvacrol, *p*-cymene, and *γ*-terpinene, all of which are present in amounts exceeding 1%. Additionally, notable hydrophilic compounds such as rosmarinic acid, 4-hydroxybenzoic acid, caffeic acid, gentisic acid, apigenin-7-glucoside, 4-hydroxybenzaldehyde, and vanillic acid have been identified [24].

In conclusion, the antimicrobial, antioxidant, and medicinal uses of *O. onites* have been investigated by numerous researchers. Extracts obtained from the leaves of the plant can be used as natural preservatives in the food industry due to their effectiveness against various pathogenic microorganisms [22]. Furthermore, the antioxidant properties of the plant may play a potential role in preventing various diseases [19]. Its medicinal uses include the treatment of respiratory infections, digestive system issues, and pain-related ailments [20]. However, there is a need for further investigation of the antimicrobial activity of *O. onites* ethanol extract (OOEt) against a wider range of microorganisms, including multidrug-resistant strains, to better understand its therapeutic potential. The primary aim of this study is to investigate the antimicrobial activity of *O. onites* ethanol extract (OOEt) against a wide range of microorganisms, including multi-drug-resistant strains, as well as its antioxidant activity, which has not been adequately studied in previous literature.

## 2. Materials and Methods

### 2.1. Plant Samples

*Origanum onites* was collected from Kazdağı (Mount Ida) Çanakkale, Türkiye (39°36′0.98″ N, 26°37′11.13″ E) and identified by Dr. Mustafa Eray Bozyel. The plant samples were placed in sample bags and transported to our laboratory. The samples were air-dried at room temperature. The voucher specimens were deposited at the Fauna and Flora Research and Application Center, Dokuz Eylül University, Buca, Izmir, Türkiye (Personel herbarium number FFDEU.Era1735).

### 2.2. Extraction

Dried *O. onites* aerial part samples were ground to obtain a fine powder and to increase the surface area for extraction. The active compounds were extracted by ethanol absolute (Sigma Aldrich, St. Louis, MO, USA) through shaking at room temperature for two days [25]. After filtering through Whatman No. 1 filter paper, the ethanol in the extract was evaporated at 45 °C under a vacuum by using a rotary evaporator (Buchi R3, BÜCHI, Labortechnik AG, Postfach, Flawil, Switzerland) [25].

### 2.3. Antibacterial and Antifungal Activity Test

The disk diffusion assay, based on Andrews’ method, was employed to assess the antibacterial and antifungal activities of OOEt [26]. Mueller-Hinton agar was poured into sterile 90-mm petri dishes to achieve a depth of approximately 4.0 ± 0.5 mm. Empty 6-mm antimicrobial susceptibility test disks were loaded with the extracts. Three different extract concentrations were obtained by loading three different volumes (namely 50 µL, 100 µL, and 200 µL) from an extract stock of 79 mg/mL onto the empty susceptibility test disks. To eliminate any potential solvent residue that could affect the results, the disks were dried at 30 °C for 24 h. The culture medium surfaces were inoculated with microorganisms suspended in a saline solution. The plates were allowed to dry for 5 min at room temperature under aseptic conditions before the disks were placed on them [25]. After incubation, the inhibitory zone sizes were measured and recorded. In the disk diffusion assay, empty sterile disks and the extraction solvent ethanol served as negative controls, while gentamicin was used as a positive control.

The Minimum Inhibitory Concentration (MIC) values of the OOEt samples were determined using the broth microdilution technique [25]. Mueller-Hinton broth (MHB) was used for cultivating various microbial strains. The cell density of each reference strain solution was adjusted to the 0.5 McFarland standard (1.5 × 10^8^ CFU/mL). A series of OOEt dilutions were prepared, and 100 µL of the sample from each dilution was transferred into 96-well sterile plates. Then, 50 µL of the microbial inocula was added to achieve a final volume of 100 µL in each well. Visual inspection was used to assess microbial growth. The positive control consisted of MHB inoculated with the test microorganisms. The MIC is the minimum concentration of OOEt necessary to inhibit bacterial growth after a 24-h incubation period. The results were reported in mg/mL following three repetitions of the tests.

### 2.4. Antioxidant Activity Test

The DPPH technique evaluates the ability of antioxidant compounds in plant extracts to scavenge DPPH radicals. To create the DPPH solution, 0.0039 g of 2,2-diphenyl-1-picrylhydrazyl (DPPH) was mixed with 50 mL of ethanol and stored in the dark until needed [27]. A 96-well plate containing DPPH solution and various concentrations of OOEt ranging from 1.075 to 200 µg/mL was incubated at room temperature for 30 min in the dark. After the incubation period, the absorbances of the wells at 515 nm were measured using a plate reader (Biotek Microplate Spectrophotometer, Winooski, VT, USA). In this experiment, ascorbic acid served as the positive control.

### 2.5. Gas Chromatography-Mass Spectroscopy Method (GC-MS)

Gas chromatography-mass spectrometry (GC-MS) is a technique utilized to separate compounds within a sample and identify their structures using mass spectrometry [28]. The Agilent 8890 GC-MS instrument (Agilent Technologies, Santa Clara, CA, USA) was employed in this study. The temperature of the injector was set to 350 °C, while helium gas was used as the carrier at a flow rate of 1 mL/min. The injector was operated in a 10:1 split mode, and the injection volume was 1 microliter. The oven temperature was programmed to increase from 40 °C to 150 °C at a rate of 4 degrees per minute, then to 180 °C at 3 degrees per minute, followed by 230 °C at 2 degrees per minute, and finally to 280 °C at 1 degree per minute. Electron ionization was used in the GC-MS technique to generate ions, which were subsequently separated based on their mass-to-charge ratios and detected. Compound identification was achieved by comparing the acquired data with the latest entries in the NIST and Wiley databases.

### 2.6. Statistics

The experiments were carried out in triplicate to ensure accuracy and reproducibility. A one-way ANOVA with a significance level of 0.05 was used for the statistical analysis. The relationship between concentration and activity was assessed using the Pearson correlation coefficient. R Studio (version 2022.12.0) was used to perform all statistical analyses.

## 3. Results

The effectiveness of OOEt in inhibiting various microorganisms can be observed through the inhibition zone diameters presented in Table 1. With different concentrations (50 µL, 100 µL, and 200 µL) tested against a wide range of bacterial and fungal strains, the data suggest that the antimicrobial activity of OOEt is concentration-dependent and exhibits varying levels of inhibition against each tested microorganism. The negative controls showed no activity, and the ANOVA test showed that there were no significant differences between parallels (*p* > 0.05) in the antimicrobial activity tests. The overall Pearson correlation coefficient for the concentration increase and the average inhibition zone diameters across about 73% of all microorganisms is higher than 0.8000, indicating a strong positive correlation (Table 2).

In order to better understand the effectiveness of OOEt against MDR strains and the resistance levels of the MDR strains used in this study, the effects of various antibiotics on these strains were investigated. The findings of this analysis are presented in Table 3, which illustrates the susceptibility of the MDR strains to a wide range of antibiotics.

The minimum inhibitory concentration (MIC) test results for a range of microorganisms, as presented in Table 4, reveal varying susceptibilities to OOEt. These microorganisms include both Gram-positive and Gram-negative bacteria, as well as yeasts. *B. subtilis* DSMZ 1971, *C. albicans* DSMZ 1386, and *L. innocua* (FI) all demonstrated MIC values of 4.28 mg/mL when exposed to OOEt. On the other hand, higher MIC values of 34.3 mg/mL were observed for *S. enteritidis* ATCC 13076, *S. typhimurium* SL 1344, *K. pneumoniae* (FI), *S. boydii* (CI), *C. tropicalis* (CI), *E. coli* (MDR), and *K. pneumoniae* (MDR) when treated with OOEt. The remaining tested microorganisms displayed MIC values between 8.57 and 17.15 mg/mL, indicating a range of susceptibilities to the OOEt.

In the DPPH radical scavenging activity test, the tested concentrations ranged from 1.075 to 200 µg/mL (Table 5). Based on the obtained results, the EC_50_ value for ascorbic acid was calculated as 8.5232 µg/mL and the EC_90_ value as 28.60 µg/mL. However, it is important to note that the lowest concentration of OOEt tested was 1.075 µg/mL, which exhibited a scavenging activity of 59.83%, surpassing the 50% threshold. Therefore, attempting to approximate the EC_50_ based on this data would result in a calculation error. Consequently, the EC_90_ calculation would also be affected by this error. Nevertheless, considering the available data, it is reasonable to suggest that the EC_90_ value for OOEt could be in the vicinity of 25 µg/mL.

**Figure 1:**
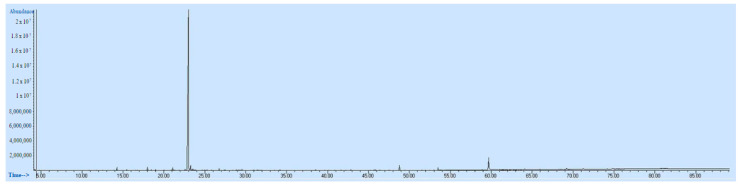
GC-MS chromatogram of OOEt.

The biochemical composition and respective percentages of OOEt, as determined by GC-MS analysis, are displayed in Table 6. The GC-MS chromatogram of OOEt is given in Figure 1.

## 4. Discussion

In this study, the antimicrobial activity of OOEt was evaluated against a variety of microorganisms using both disk diffusion and MIC methods. OOEt demonstrated antimicrobial activity against all 30 tested strains, with high susceptibility (≥15 mm) in each instance where 200 mL of the extract was applied. In the disk diffusion test, the most susceptible Gram-positive bacterium, *E. faecium*, showed a 52-mm inhibition zone at 100 µL of OOEt and a MIC value of 17.15 mg/mL. Among Gram-negative bacteria, *P. aeruginosa* and *P. fluorescens* displayed the highest sensitivity, both presenting a 24 mm inhibition zone at 100 µL of OOEt in the disk diffusion test and a MIC value of 17.15 mg/mL. For multidrug-resistant bacteria, *E. coli* (MDR) exhibited the highest susceptibility compared to all positive controls, with a disk diffusion inhibition zone of 19 mm at 200 µL of OOEt and a MIC value of 34.3 mg/mL. In the case of fungal strains, OOEt was more effective than the positive controls, with inhibition zones observed in the disk diffusion test and corresponding MIC values.

*A. baumannii* is an opportunistic pathogen that colonizes hospitalized patients, leading to severe infections, septic shock, and death. These bacteria often cause urinary tract infections and pneumonia, especially in patients in intensive care units [44]. A large-scale surveillance study conducted in the United States found that *A. baumannii* is responsible for 5–10% of acquired cases of pneumonia in intensive care [45]. Although the frequency of nosocomial pneumonia caused by *A. baumannii* varies from country to country and region to region (27–50%), the mortality rate in these types of pneumonia is between 30 and 70% [46]. Among *A. baumannii* infections, urinary tract infections experienced by patients with catheters have an important place. As a result of a study, it was found that 1.6% of urinary tract infections acquired in intensive care were due to *A. baumannii* [47]. Cases of meningitis associated with *A. baumannii* also occur, especially in patients undergoing brain surgery with ventricular drainage. The mortality rates (70%) of these cases are quite high [48]. In addition, these microorganisms lead to many types of infections, such as skin and wound infections, endocarditis, peritonitis (often in patients with peritoneal dialysis), conjunctivitis, osteomyelitis, and synovitis [44]. Bacteremia and sepsis caused by *A. baumannii* are also common in patients in intensive care units [44,46]. The widespread use of broad-spectrum antibiotics in hospitals has led to the rapid emergence of multidrug-resistant (MDR) strains of *A. baumannii*. Despite this, only a few antibiotics are effective against *A. baumannii* (MDR) infections [49]. Ozgen et al. [50] observed a 10.5 mm inhibition zone for the ethanol extract of *O. vulgare* leaves against *A. baumannii* ATCC BAA-747. Canlı et al. [51] showed that *Lavandula stoechas* (Lamiaceae) caused 11 mm of inhibition zone for 35.1 mg ethanol extract against the same *A. baumannii* (MDR) strain. In our study, we determined that OOEt presented a 19 mm inhibition zone for 200 µL OOEt against *A. baumannii* (MDR) and a MIC value of 34.3 mg/mL. This result is proof that OOEt Is more effective than the other two plants.

In our study, the *A. baumannii* (MDR) strain we used demonstrated high resistance, with the largest measured zone being 16 mm for the tested antibiotics, indicating that most antibiotics were ineffective or displayed very low efficacy. However, our findings indicate that OOEt effectively inhibits the growth of *A. baumannii* (MDR), highlighting the potential of OOEt as a promising candidate for the development of new antimicrobial agents, especially against highly resistant strains such as the *A. baumannii* (MDR) strain used in our study.

Enterococci are facultative anaerobic Gram-positive bacteria that naturally inhabit the intestinal flora of animals and humans. These bacteria are typically considered to have low pathogenicity, mainly infecting immunocompromised individuals in oncology, hematology, nephrology, or transplantation units. Enterococci can cause various infections in the urinary and biliary tracts, wounds, and life-threatening diseases, such as bacteremia or endocarditis. They are the second- to third-most important bacterial group, causing approximately 12% of nosocomial infections [52]. The *Enterococcus* genus comprises over 50 species, with *E. faecalis* and *E. faecium* being the primary causative agents of infections in humans. Enterococci emerged in the 1970s as a leading cause of nosocomial infections [53]. *E. faecalis* accounts for 85–90% of enterococcal infections, while *E. faecium* accounts for 5–10% [54]. In the last two decades, *E. faecium* has rapidly evolved as a global nosocomial pathogen, successfully adapting to the nosocomial environment and acquiring resistance to glycopeptides [53]. Sener et al. [55] reported the antimicrobial activity of a 65% ethanol extract of *Origanum majorana* against fifteen bacterial strains, including *E. faecium*. The extract showed antimicrobial activity against *E. faecium* with a 9-mm inhibition zone at 100 µL. In our study, we found that OOEt had a 52-mm inhibition zone at 100 µL against *E. faecium* and a MIC value of 21.7 mg/mL. These results indicate that OOEt is more effective against *E. faecium* than the ethanol extract of *O. majorana*.

*Pseudomonas aeruginosa* (Pseudomonadaceae) is a Gram-negative bacterium that is ubiquitous and can survive in a wide variety of environments [56]. *P. aeruginosa*, defined as an opportunistic pathogen, is the most common bacterium that causes nosocomial infections, bacteremia, ventilator-associated pneumonia, urinary tract infections, and skin and soft tissue infections [56,57]. *P. aeruginosa* causes fatal infections in immunocompromised patients in oncology, post-surgery, severe burns, or those infected by HIV. It has been described as one of the most life-threatening bacteria and was listed by the WHO as a priority pathogen in the R&D of new antibiotics in 2017. Due to the adaptability of *P. aeruginosa* and high antibiotic resistance, antibiotics often show limited efficacy, and thus mortality increases [58]. Husein et al. [59] observed a 14.7-mm inhibition zone for a 70% ethanol extract of *Origanum syriacum* against *P. aeruginosa*. In our study, we found that OOEt exhibited a 24 mm inhibition zone in the disk diffusion assay using 100 µL and had a MIC value of 17.15 mg/mL against *P. aeruginosa*. These results showed that OOEt has more effective results compared to the previously reported *O. syriacum* extract, highlighting its potential in terms of antimicrobial efficacy against *P. aeruginosa*.

*Candida* species are among the most deadly fungi. *Candida* species cause invasive candidiasis in immunocompromised patients who have been in intensive care for a long time due to severe trauma. Among them, *C. albicans* is the most common cause of life-threatening systemic candidiasis. *C. albicans* is an opportunistic pathogen that exists symbiotically in most individuals and is one of the most common causes of mucosal and systemic infections. *C. albicans*, unlike most fungal pathogens, is generally considered to be obligatorily associated with warm-blooded animals [60,61]. Kerbouche et al. [62] discovered the antimicrobial activity of an ethanol extract of *Origanum floribundum* against *C. albicans* with a 9.7 mm inhibition zone. In our study, we found that OOEt exhibited a 15-mm inhibition zone in the disk diffusion assay using 200 µL against *C. albicans*, demonstrating more effective results compared to the previously reported *O. floribundum* extract. Furthermore, the MIC value of OOEt was found to be 4.28 mg/mL, which highlights its notably high efficacy as an antimicrobial agent against *C. albicans*. Additionally, our results also revealed significant antimicrobial activity against another *Candida* species, *C. tropicalis*, with inhibition zones of 31 mm for both 100 and 200 µL and a MIC value of 34.3 mg/mL, emphasizing the importance of OOEt as a potential antimicrobial agent against multiple *Candida* species.

Living organisms are constantly exposed to reactive oxygen species generated as a result of respiratory, metabolic, or disease stress [63]. It is important to eliminate oxidation caused by reactive oxygen species, which cause many diseases, and to neutralize free radicals [64]. In our study, we observed that the DPPH radical scavenging activity of OOEt was comparable to that of ascorbic acid, which served as a positive control. The EC_50_ value for ascorbic acid was determined to be 8.5232 µg/mL, while the EC_90_ value was found to be 28.60 µg/mL. Based on our findings, we propose that the EC_90_ value for OOEt is approximately 25 µg/mL, which falls within or below the EC_90_ range of ascorbic acid. In a study conducted by Kosakowska et al. [65], the antioxidant activity of essential oils and hydroethanolic extracts from Greek oregano (*O. vulgare* L. subsp. *hirtum*) and common oregano (*O. vulgare* L. subsp. *vulgare*) was evaluated. The DPPH scavenging activities for the hydroethanolic extracts of Greek and common oregano were reported as 70.90% and 69.83%, respectively, with corresponding Trolox equivalent values of 252.10 and 242.43 µmol/g. In another study by Kaurinovic et al. [66], the DPPH scavenging activity of various *O. basilicum* and *O. vulgare* extracts was investigated, and the IC_50_ values for *O. vulgare* water and n-BuOH extracts were found to show stronger antioxidant effects than BHT. In comparison to the findings of Kaurinovic et al., our results indicate that OOEt possesses a more potent antioxidant capacity. Our findings demonstrate that OOEt has remarkably potent antioxidant activity, even surpassing the effect of ascorbic acid, a well-known antioxidant agent. This suggests that OOEt could be a valuable natural source of antioxidants and may have potential applications in the prevention and treatment of diseases associated with oxidative stress.

The GC-MS analysis of OOEt revealed the presence of several compounds with known biological activities, which may contribute to the observed antimicrobial and antioxidant activities. The most abundant compound identified in the extract was carvacrol (82.34%), which has been previously reported to exhibit antioxidant and antimicrobial activities [34,35,36]. Other notable compounds include thymoquinone (1.09%), which has demonstrated neuroprotective and anti-inflammatory effects [32,33], and borneol (1.00%), which has been shown to possess antibacterial activity [30]. In addition to these major compounds, the extract also contained several other biologically active compounds, albeit in smaller quantities. These include sabinene hydrate (0.75%), which has been reported to have antioxidant activity [29], 4-carvomenthenol (0.79%), known for its anti-inflammatory activity [31], thymol (0.38%), which has antioxidant and antimicrobial activities [34,35,36], carvacrol acetate (0.15%), which has anti-inflammatory and anti-nociceptive activities [37], and caryophyllene (0.63%), known for its antibiofilm and anticancer activities [38,39]. Furthermore, β-bisabolene (0.39%) has been shown to exhibit anticancer and bactericidal activities [40,41], and eicosane (0.66%) has demonstrated antifungal activity [42]. The presence of these bioactive compounds in OOEt, particularly in high quantities, such as carvacrol, may help explain the potent antimicrobial and antioxidant activities observed in our study. The synergistic effects of these compounds could also contribute to the overall efficacy of OOEt as a potential natural antimicrobial and antioxidant agent.

In summary, our study demonstrated that OOEt exhibits potent antimicrobial activity against a wide range of pathogenic microorganisms, including both bacterial and fungal strains. In some cases, the antimicrobial efficacy of OOEt was even more potent than that of synthetic antibiotics, highlighting its potential as a natural alternative for combating infections. Additionally, the antioxidant activity of OOEt was found to be stronger than that of ascorbic acid, a widely used antioxidant compound. These findings suggest that OOEt may serve as a valuable natural source of antimicrobial and antioxidant agents, which could be beneficial for various applications in medicine, food preservation, and cosmetics. Further studies are warranted to explore the potential synergistic effects of the bioactive compounds identified in OOEt as well as to investigate their safety and efficacy in vivo.

## Figures and Tables

**Table 1:** Disk diffusion test results of OOEt (inhibition zone diameters in mm).

No	Microorganisms	50 **µL ^1^**	100 **µL ^1^**	200 **µL ^1^**	Gen	Amp	Tob
1	*Bacillus subtilis* DSMZ 1971	17.00 ± 0.00	19.00 ± 0.00	27.00 ± 0.00	30	41	26
2	*Candida albicans* DSMZ 1386	12.00 ± 0.00	15.00 ± 0.58	17.00 ± 0.00	12	0	13
3	*Enterobacter aerogenes* ATCC 13048	10.00 ± 0.00	14.00 ± 0.00	17.00 ± 0.00	24	0	18
4	*Enterococcus faecalis* ATCC 29212	11.00 ± 0.00	14.00 ± 0.00	18.00 ± 0.58	12	14	8
5	*Escherichia coli* ATCC 25922	10.00 ± 0.00	14.00 ± 0.00	18.00 ± 0.00	22	6	20
6	*Listeria monocytogenes* ATCC 7644	10.00 ± 0.00	13.00 ± 0.00	28.00 ± 0.00	28	23	24
7	*Pseudomonas aeruginosa* DSMZ 50071	18.00 ± 0.00	24.00 ± 0.00	15.00 ± 0.00	15	0	22
8	*Pseudomonas fluorescens* P1	19.00 ± 0.58	24.00 ± 0.00	18.00 ± 0.00	13	14	12
9	*Salmonella enteritidis* ATCC 13076	11.00 ± 0.00	16.00 ± 0.00	19.00 ± 0.00	21	16	15
10	*Salmonella typhimurium* SL 1344	11.00 ± 0.00	12.00 ± 0.00	21.00 ± 0.00	24	13	15
11	*Staphylococcus aureus* ATCC 25923	20.00 ± 0.00	23.00 ± 0.00	36.00 ± 0.58	21	25	14
12	*Staphylococcus epidermidis* DSMZ 20044	18.00 ± 0.00	19.00 ± 0.00	29.00 ± 0.00	22	24	20
13	*Enterococcus durans* (FI)	12.00 ± 0.00	16.00 ± 0.00	20.00 ± 0.00	11	28	13
14	*Enterococcus faecium* (FI)	50.00 ± 0.00	52.00 ± 0.00	42.00 ± 0.00	28	32	15
15	*Klebsiella pneumoniae* (FI)	12.00 ± 0.00	15.00 ± 1.15	16.00 ± 0.00	19	6	23
16	*Listeria innocua* (FI)	11.00 ± 0.00	13.00 ± 0.00	19.00 ± 0.58	13	13	15
17	*Salmonella infantis* (FI)	11.00 ± 0.00	13.00 ± 0.00	19.00 ± 0.00	17	14	14
18	*Salmonella kentucky* (FI)	11.00 ± 0.00	15.00 ± 0.00	20.00 ± 0.00	12	15	16
19	*Escherichia coli* (FI)	11.00 ± 0.00	16.00 ± 0.00	16.00 ± 0.00	20	0	0
20	*Staphylococcus aureus* (CI)	25.00 ± 0.00	26.00 ± 0.00	36.00 ± 0.00	22	0	18
21	*Shigella boydii* (CI)	11.00 ± 0.00	17.00 ± 0.00	17.00 ± 0.00	20	0	18
22	*Candida tropicalis* (CI)	28.00 ± 0.00	31.00 ± 0.00	31.00 ± 0.00	0	0	0
23	*Escherichia coli* (MDR)	10.00 ± 0.00	16.00 ± 0.00	19.00 ± 0.00	8	0	9
24	*Klebsiella pneumoniae* (MDR)	11.00 ± 0.00	16.00 ± 0.00	14.00 ± 0.00	15	8	20
25	*Acinetobacter baumannii* (MDR)	12.00 ± 0.00	17.00 ± 0.00	19.00 ± 0.00	0	0	0
26	*Enterobacter aerogenes* (MDR)	11.00 ± 0.00	11.00 ± 0.00	15.00 ± 0.00	16	0	18
27	*Serratia odorifera* (MDR)	10.00 ± 0.00	15.00 ± 0.00	19.00 ± 0.00	7	0	9
28	*Proteus vulgaris* (MDR)	11.00 ± 0.00	14.00 ± 0.00	18.00 ± 0.00	11	9	11
29	*Streptococcus pneumoniae* (MDR)	11.00 ± 0.00	13.00 ± 0.00	18.00 ± 0.00	10	9	8
30	*Staphylococcus aureus* (MRSA + MDR)	29.00 ± 0.00	29.00 ± 1.15	29.00 ± 0.00	22	22	21

^1^ The data are given as the mean values of three replicates with standard errors; CI: Clinical isolated; FI: Food isolated; MDR: Multidrug-resistant; MRSA: Methicillin-resistant *S. aureus*; Gen: Gentamicin; Amp: Ampicillin; Tob: Tobramycin.

**Table 2:** Pearson correlation test results and 95% confidence interval for the differences of means for the disk diffusion test results of OOEt.

	Pearson Correlation Test		Confidence Interval for the Differences in Means		
Microorganisms	Correlation	*p*-Value	EMM ^1^	Lower CL ^2^	Upper CL ^3^
*Bacillus subtilis* DSMZ 1971	0.9897	0.0913	21.0	15.79	26.2
*Candida albicans* DSMZ 1386	0.9538	0.1942	14.7	9.46	19.9
*Enterobacter aerogenes* ATCC 13048	0.9631	0.1734	13.7	8.46	18.9
*Enterococcus faecalis* ATCC 29212	0.9942	0.0686	14.3	9.12	19.5
*Escherichia coli* ATCC 25922	0.9820	0.1210	14.0	8.79	19.2
*Listeria monocytogenes* ATCC 7644	0.9843	0.1129	17.0	11.79	22.2
*Pseudomonas aeruginosa* DSMZ 50071	−0.5000	0.6667	19.0	13.79	24.2
*Pseudomonas fluorescens* P1	−0.3394	0.7795	20.3	15.12	25.5
*Salmonella enteritidis* ATCC 13076	0.9449	0.2123	15.3	10.12	20.5
*Salmonella typhimurium* SL 1344	0.9707	0.1544	14.7	9.46	19.9
*Staphylococcus aureus* ATCC 25923	0.9878	0.0994	26.3	21.12	31.5
*Staphylococcus epidermidis* DSMZ 20044	0.9686	0.1599	22.0	16.79	27.2
*Enterococcus durans* (FI)	0.9820	0.1210	16.0	10.79	21.2
*Enterococcus faecium* (FI)	−0.8660	0.3333	48.0	42.79	53.2
*Klebsiella pneumoniae* (FI)	0.8910	0.3000	14.3	9.12	19.5
*Listeria innocua* (FI)	0.9960	0.0579	14.3	9.12	19.5
*Salmonella infantis* (FI)	0.9960	0.0579	14.3	9.12	19.5
*Salmonella kentucky* (FI)	0.9921	0.0803	15.3	10.12	20.5
*Escherichia coli* (FI)	0.7559	0.4544	14.3	9.12	19.5
*Staphylococcus aureus* (CI)	0.9686	0.1599	29.0	23.79	34.2
*Shigella boydii* (CI)	0.7559	0.4544	15.0	9.79	20.2
*Candida tropicalis* (CI)	0.7559	0.4544	30.0	24.79	35.2
*Escherichia coli* (MDR)	0.9286	0.2421	15.0	9.79	20.2
*Klebsiella pneumoniae* (MDR)	0.4336	0.7145	13.7	8.46	18.9
*Acinetobacter baumannii* (MDR)	0.9078	0.2755	16.0	10.79	21.2
*Enterobacter aerogenes* (MDR)	0.9449	0.2123	12.3	7.12	17.5
*Serratia odorifera* (MDR)	0.9679	0.1618	14.7	9.46	19.9
*Proteus vulgaris* (MDR)	0.9942	0.0687	14.3	9.12	19.5
*Streptococcus pneumoniae* (MDR)	0.9986	0.0334	14.0	8.79	19.2
*Staphylococcus aureus* (MRSA + MDR)	NA	NA	29.0	23.79	34.2

^1^ Estimated Marginal Mean; ^2^ The lower bound of the confidence interval; ^3^ The upper bound of the confidence interval.

**Table 3:** Antibiotic Susceptibility Test for MDR Strains (inhibition zone diameters in mm).

Antibiotic	Ec	Kp	Ab	Ee	So	Pv	Sp	Sa
Gentamicin	8	15	-	16	7	11	10	22
Tobramycin	9	20	-	18	9	11	8	21
Ciprofloxacin	7	21	-	32	23	42	42	27
Cefazolin	-	-	-	11	-	-	-	26
Clindamycin	-	-	-	-	-	9	9	38
Chloramphenicol	26	25	9	31	28	22	22	30
Ceftriaxone	-	22	-	32	8	23	26	19
Ampicillin	8	8	8	-	-	9	9	22
Cephalothin	-	-	-	-	-	-	-	28
Cefuroxime	-	9	-	18	-	20	19	31
Vancomycin	8	-	8	-	8	-	9	19
Amoxicillin/Clavulanic acid	12	-	-	-	13	9	10	25
Trimethoprim/Sulfamethoxazole	-	-	-	30	-	30	8	30
Clarithromycin	-	8	-	15	-	10	10	15
Aztreonam	9	29	-	33	16	37	40	-
Piperacillin/Tazobactam	20	27	-	15	22	32	31	23
Ampicillin/Sulbactam	8	-	-	-	10	12	15	23
Ceftazidime	12	15	-	31	21	25	27	19
Rifampicin	-	-	10	8	9	13	11	36
Oxacillin	-	-	-	8	-	-	-	17
Piperacillin	-	14	-	-	8	24	24	21
Linezolid	-	-	-	-	-	11	13	33
Teicoplanin	8	8	-	8	8	8	9	18
Amikacin	20	25	8	29	18	26	29	25
Polymyxin B	16	15	16	14	14	12	10	9
Cefoxitin	8	8	8	-	19	12	10	20
Imipenem	34	27	9	28	29	26	30	56
Sulbactam/Cefoperazone	10	13	-	9	10	16	13	20
Colistin sulfate	14	20	13	13	10	-	9	8
Furazolidone	29	28	10	25	23	12	13	17
Optochin	8	8	-	8	8	8	8	-
Bacitracin	-	8	-	8	-	-	-	8
Cefotaxime	-	19	-	30	-	22	14	22

Ec: *E. coli*; Kp: *K. pneumoniae*; Ab: *A. baumannii*; Ee: *E. aerogenes*; So: *S. odorifera*; Pv: *P. vulgaris*; Sp: *S. pneumoniae*; Sa: *S. aureus*.

**Table 4:** Minimum inhibitory concentration (MIC) test results.

No	Microorganisms	MIC (mg/mL)
1	*Bacillus subtilis* DSMZ 1971	4.28
2	*Candida albicans* DSMZ 1386	4.28
3	*Enterobacter aerogenes* ATCC 13048	17.15
4	*Enterococcus faecalis* ATCC 29212	17.15
5	*Escherichia coli* ATCC 25922	17.15
6	*Listeria monocytogenes* ATCC 7644	8.57
7	*Pseudomonas aeruginosa* DSMZ 50071	17.15
8	*Pseudomonas fluorescens* P1	17.15
9	*Salmonella enteritidis* ATCC 13076	34.3
10	*Salmonella typhimurium* SL 1344	34.3
11	*Staphylococcus aureus* ATCC 25923	17.15
12	*Staphylococcus epidermidis* DSMZ 20044	17.15
13	*Enterococcus durans* (FI)	8.57
14	*Enterococcus faecium* (FI)	17.15
15	*Klebsiella pneumoniae* (FI)	34.3
16	*Listeria innocua* (FI)	4.28
17	*Salmonella infantis* (FI)	17.15
18	*Salmonella kentucky* (FI)	17.15
19	*Escherichia coli* (FI)	8.57
20	*Staphylococcus aureus* (CI)	17.15
21	*Shigella boydii* (CI)	34.3
22	*Candida tropicalis* (CI)	34.3
23	*Escherichia coli* (MDR)	34.3
24	*Klebsiella pneumoniae* (MDR)	34.3
25	*Acinetobacter baumannii* (MDR)	17.15
26	*Enterobacter aerogenes* (MDR)	8.57
27	*Serratia odorifera* (MDR)	8.57
28	*Proteus vulgaris* (MDR)	17.15
29	*Streptococcus pneumoniae* (MDR)	17.15
30	*Staphylococcus aureus* (MRSA + MDR)	17.15

**Table 5:** DPPH radical scavenging activity results for OOEt and ascorbic acid (%).

Concentrations (µg/mL)	OOEt (%)	Ascorbic Acid (%)
200.000	99.34	94.67
100.000	98.66	93.39
50.000	93.11	92.08
25.000	90.08	90.09
12.500	90.62	69.94
6.250	88.75	35.79
3.125	88.65	17.70
1.075	59.83	8.74

**Table 6:** GC-MS analysis of OOEt.

No	RT	Chemical Structures	Compound Name	Formula	MW (g/mol)	Area (%)	Known Activity
1	14.269	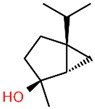	Sabinene hydrate	C_10_H_18_O	154.249	0.75	Antioxidant activity [29]
2	15.487	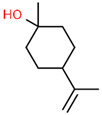	β -Terpineol	C_10_H_18_O	154.249	0.26	-
3	18.004	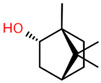	Borneol	C_10_H_18_O	154.249	1.00	Antibacterial activity [30]
4	18.451	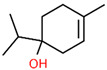	4-Carvomenthenol	C_10_H_18_O	154.249	0.79	Anti-inflammatory activity [31]
5	21.089	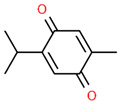	Thymoquinone	C_10_H_12_O_2_	164.201	1.09	Neuroprotective and Anti-inflammatory effects [32,33]
6	22.597	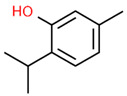	Thymol	C_10_H_14_O	150.218	0.38	Antioxidant and antimicrobial activity [34,35,36]
7	23.003	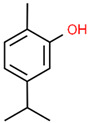	Carvacrol	C_10_H_14_O	150.218	82.34	Antioxidant and antimicrobial activity [34,35,36]
8	23.192	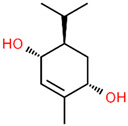	2-Methyl-5-(propan-2-ylidene)cyclohexane-1,4-diol	C_10_H_18_O_2_	170.249	2.49	-
9	25.261	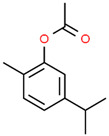	Carvacrol acetate	C_12_H_16_O_2_	192.254	0.15	Anti-inflammatory and anti-nociceptive activity [37]
10	26.738	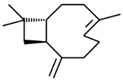	Caryophyllene	C_15_H_24_	204.351	0.63	Antibiofilm and anticancer activity [38,39]
11	29.504	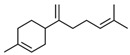	β-Bisabolene	C_15_H_24_	204.351	0.39	Anticancer and bactericidal activity [40,41]
12	38.574	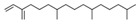	Neophytadiene	C_20_H_38_	278.516	0.38	-
13	45.801	-	Unknown	-	-	1.79	-
14	53.518	-	Unknown	-	-	1.00	-
15	59.292	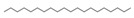	Eicosane	C_20_H_42_	282.547	0.66	Antifungal activity [42]
16	59.698	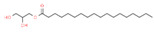	Glyceryl Monostearate	C_21_H_42_O_4_	358.556	5.64	Antibacterial activity [43]
17	64.101	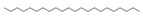	Docosane	C_22_H_46_	310.601	0.34	-
18	65.957	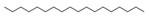	Octadecane	C_18_H_38_	254.494	0.25	-

Figures: https://pubchem.ncbi.nlm.nih.gov/, http://www.chemspider.com/ (accessed on 27 April 2023).

## Data Availability

All data that were generated or analyzed during this study have been included in this published article.
